# An analysis of AURKB's prognostic and immunological roles across various cancers

**DOI:** 10.1111/jcmm.18475

**Published:** 2024-06-19

**Authors:** Jun Li, Cui Cheng, Jiajun Zhang

**Affiliations:** ^1^ Department of Urology The First Affiliated Hospital of Bengbu Medical University Bengbu China; ^2^ Department of Gynaecological Oncology The First Affiliated Hospital of Bengbu Medical University Bengbu China

**Keywords:** biomarker, cell cycle, immune infiltration, immunotherapy, kidney renal clear cell carcinoma, pan‐cancer

## Abstract

Aurora kinase B (AURKB), an essential regulator in the process of mitosis, has been revealed through various studies to have a significant role in cancer development and progression. However, the specific mechanisms remain poorly understood. This study, therefore, seeks to elucidate the multifaceted role of AURKB in diverse cancer types. This study utilized bioinformatics techniques to examine the transcript, protein, promoter methylation and mutation levels of AURKB. The study further analysed associations between AURKB and factors such as prognosis, pathological stage, biological function, immune infiltration, tumour mutational burden (TMB) and microsatellite instability (MSI). In addition, immunohistochemical staining data of 50 cases of renal clear cell carcinoma and its adjacent normal tissues were collected to verify the difference in protein expression of AURKB in the two tissues. The results show that AURKB is highly expressed in most cancers, and the protein level of AURKB and the methylation level of its promoter vary among cancer types. Survival analysis showed that AURKB was associated with overall survival in 12 cancer types and progression‐free survival in 11 cancer types. Elevated levels of AURKB were detected in the advanced stages of 10 different cancers. AURKB has a potential impact on cancer progression through its effects on cell cycle regulation as well as inflammatory and immune‐related pathways. We observed a strong association between AURKB and immune cell infiltration, immunomodulatory factors, TMB and MSI. Importantly, we confirmed that the AURKB protein is highly expressed in kidney renal clear cell carcinoma (KIRC). Our study reveals that AURKB may be a potential biomarker for pan‐cancer and KIRC.

## 1 INTRODUCTION

According to the 2020 statistics provided by the International Agency for Research on Cancer, there has been an increasing incidence of cancer each year, constituting a substantial threat to both public health and the economy.^1^ Current cancer treatment modalities include surgical intervention, chemotherapy and radiation therapy. Additionally, targeted therapy and immunotherapy are emerging as innovative strategies in the battle against cancer. Despite some clinical successes, targeted therapy and immunotherapy are often hampered by factors such as negative drug targets or immune checkpoints, the development of tumour resistance and severe toxic side effects.^2^, ^3^, ^4^ As a result, the ongoing search for novel markers and therapeutic targets that are highly specific or sensitive is of paramount importance in contemporary cancer research.^5^

The complexity of cancer development involves multifaceted stages and levels, including the activation of oncogenes, inactivation of tumour suppressor genes, genomic instability, alterations in epigenetic regulation and disruptions in cell signalling pathways. Cumulatively, these factors contribute to the synthesis of abnormal proteins and stress signals.^6^, ^7^ A growing body of evidence points to the occurrence of abnormal cell division, characterized by the formation of cells with an atypical number of chromosomes, as a factor that may enhance cancer progression.^8^, ^9^ Mitosis, the crucial process upon which cell division relies, is regulated by key components such as Aurora kinase B (AURKB). Aberrations in AURKB, such as overexpression, mutation, or amplification, may lead to anomalous cell division and the creation of aneuploid cells with irregular chromosome count and structure. These alterations consequently elevate the likelihood of genetic mutations and enhance susceptibility to carcinogenesis.^10^ Moreover, the AURKB gene has been validated as an actively involved oncogene in cancer and displays a significant inverse correlation with cancer prognosis.^11^ Specific roles of AURKB in certain cancer types, including non‐small cell lung cancer (NSCLC)^12^ and prostate cancer,^13^ have been explored in prior studies. Nevertheless, a more exhaustive examination of the molecular mechanisms underlying AURKB's role is essential for the development of innovative pathways in cancer diagnosis and therapeutic research.

**FIGURE 1:**
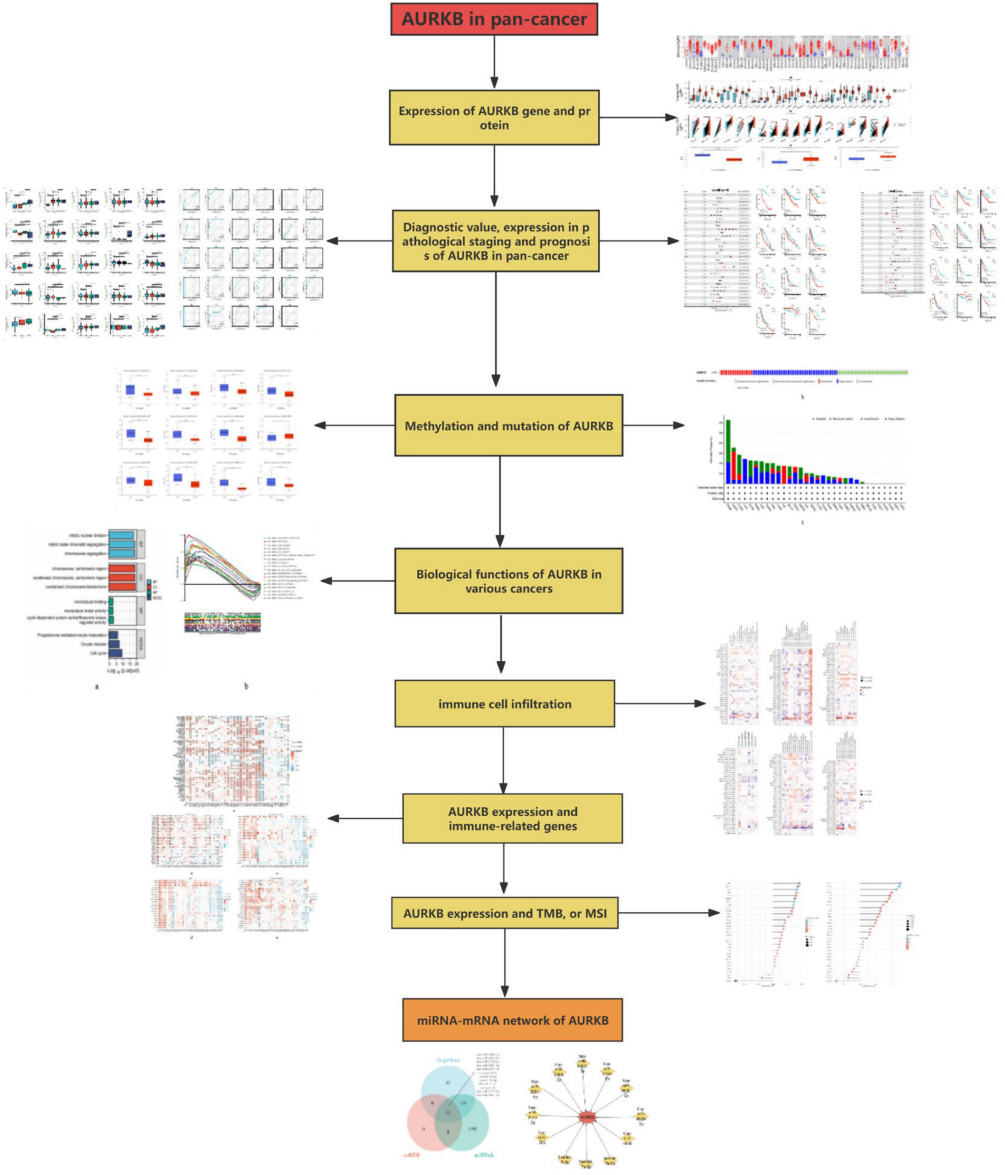
Flow chart of this study.

Our study delved into the expression pattern of AURKB in order to assess its significance, genetic alterations and epigenetic modifications in the context of pan‐cancer diagnosis and prognosis. Furthermore, we examined the correlation between AURKB expression levels, immune cell infiltration in pan‐cancer tissues, immune‐related gene expression, as well as tumour mutational burden (TMB) and microsatellite instability (MSI). Additionally, we conducted enrichment analysis of genes co‐expressed with AURKB to elucidate the potential biological functions and oncogenic mechanisms associated with AURKB. Furthermore, our investigation delved into the miRNA‐AURKB regulatory network, uncovering the potential utility of AURKB as a diagnostic, prognostic and predictive target for anti‐cancer therapy across various types of cancer. This novel finding is illustrated in Figure 1, depicting the workflow of our study.

## 2 METHODS

### 2.1 Gathering and analysing information

The study investigated the expression of AURKB in various cancers and normal tissues using normal tissue data from the Genote‐Tissue Expression (GTEx) database and cancer genomic data from The Cancer Genome Atlas (TCGA). The downloaded data included RNA sequencing data and clinical follow‐up information for patients with 33 types of cancer. A log2 conversion was employed to normalize all expression data. Cancers include adrenocortical carcinoma (ACC), bladder urothelial carcinoma (BLCA), breast invasive carcinoma (BRCA), cervical squamous cell carcinoma and endocervical adenocarcinoma (CESC), cholangiocarcinoma (CHOL), colon adenocarcinoma (COAD), lymphoid neoplasm diffuse large B‐cell lymphoma (DLBC), oesophageal carcinoma (ESCA), glioblastoma multiforme (GBM), head and neck squamous cell carcinoma (HNSC), kidney chromophobe (KICH), kidney renal clear cell carcinoma (KIRC), kidney renal papillary cell carcinoma (KIRP), acute myeloid leukaemia (LAML), brain lower grade glioma (LGG), liver hepatocellular carcinoma (LIHC), lung adenocarcinoma (LUAD), lung squamous cell carcinoma (LUSC), Mesothelioma (MESO), ovarian serous cystadenocarcinoma (OV), pancreatic adenocarcinoma (PAAD), pheochromocytoma and paraganglioma (PCPG), prostate adenocarcinoma (PRAD), rectum adenocarcinoma (READ), sarcoma (SARC), skin cutaneous melanoma (SKCM), stomach adenocarcinoma (STAD), testicular germ cell tumours (TGCT), thyroid carcinoma (THCA), thymoma (THYM), uterine corpus endometrial carcinoma (UCEC), uterine carcinosarcoma (UCS) and uveal melanoma (UVM).

### 2.2 Analysis of AURKB expression

The mRNA expression level of AURKB was assessed utilizing the Tumour Immunity Estimation Resource (TIMER) 2.0 database (http://timer.cistrome.org/) and R software. Additionally, the Clinical Proteomic Tumour Analysis Consortium (CPTAC) database was leveraged to evaluate the abundance of AURKB protein.

### 2.3 Analysing the diagnostic value of AURKB

Receiver operating characteristic (ROC) curves were employed to analyse the diagnostic accuracy of AURKB in distinct cancer types. The area under the cure (AUC) ranged from 0.5 (indicating no diagnostic value) to 1.0 (representing exceptional diagnostic value). An AUC exceeding 0.9 was considered indicative of high diagnostic value.

### 2.4 The methylation levels and genetic changes in pan‐cancer associated with AURKB

The study examined AURKB promoter methylation in cancers using the UALCAN database (http://ualcan.path.uab.edu/). The cBioPortal database (http://www.cbioportal.org/) was implemented to identify genetic changes in AURKB within the TCGA pan‐cancer dataset. The modules utilized to access gene alteration data for AURKB included ‘Oncoprint,’ ‘Cancer Type Summary,’ and ‘Mutations.’

### 2.5 The relation between AURKB expression and prognosis as well as pathological stage

The relevance between the prognosis of patients' expression and AURKB mRNA was estimated using Kaplan–Meier and Cox proportional hazard models. This study examined the survival outcomes of 33 various types of cancer, encompassing both overall survival (OS) and disease‐specific survival (DSS). The results were represented by Kaplan–Meier curves and forest plots. An efficient and practical approach to estimate OS for specific patients was achieved by selecting KIRC datasets with sample sizes greater than 500. The precision of the nomogram was evaluated by generating calibration curves for predicting outcomes within the upcoming 1, 3 and 5 years.

### 2.6 Functional enrichment analysis of AURKB

We identified 20 genes associated with AURKB expression in *Homo sapiens* from the STRING database (https://cn.string‐db.org). To explore the function of AURKB in cancers, we utilized Gene Ontology (GO) and Kyoto Encyclopedia of Genes and Genomes (KEGG) analyses. To further investigate AURKB's possible functions, we categorized AURKB expression profiles into two groups: high AURKB expression and low AURKB expression, using the median AURKB expression as the cut‐off point. The potential molecular pathways of AURKB in KIRC were determined through genome enrichment analysis (GSEA). Genomes with |NES| > 1, NOM *p* < 0.05 and FDR *q* < 0.25 were considered significantly enriched according to the GSEA analysis's HALLMARK.

### 2.7 Pan‐cancer examination of AURKB expression in relation to immune modulation and immune cell infiltration

The TIMER 2.0 database employed TIMER, EPIC, TIDE and other algorithms to evaluate the correlation between immune cell infiltration and AURKB expression in pan‐cancer. Immune cells such as B cells, CD4^+^ T cells, CD8^+^ T cells, neutrophils, macrophages and dendritic cells were analysed. Additionally, we examined the co‐expression of AURKB and immune‐related genes, including the major histocompatibility complex (MHC), immune activation, immunosuppression, chemokines and chemokine receptors. The results are presented using heat maps.

### 2.8 The relationship between AURKB and TMB or MSI

We investigated the relationship between AURKB expression and TMB or MSI employing Spearman's methodology. The results are displayed through bar graphs.

### 2.9 Target miRNA prediction

We obtained the target miRNAs of AURKB from three prediction databases, including miRDB (http://mirdb.org/miRDB/), TargetScan (https://www.targetscan.org/vert_72/) and miRWalk (http://mirwalk.umm.uni‐heidelberg.de/) databases, respectively and then obtained the target miRNAs common to the three databases, and finally built a network graph of AURKB with the target miRNAs.

### 2.10 Immunohistochemical (IHC) analysis

The Department of Pathology of the First Affiliated Hospital of Bengbu Medical College, Bengbu, Anhui Province, China, provided postoperative specimen paraffin of 50 patients with renal clear cell carcinoma, including the paraffin sections of 50 cancerous and 33 paracarcinogenic tissue specimens. AURKB antibody was purchased from Hangzhou Ziran Biologicals Company (Article number: ab45145). The Ethics Committee of the First Affiliated Hospital of Bengbu Medical College approved this study (Ethical Approval Number: 2023YJS037).

The prepared paraffin tissue sections were baked at 65°C for 1 h and then sequentially subjected to xylene dewaxing, gradient ethanol dehydration and high‐temperature repair of antigens.After rinsing with PBS buffer, the sections were incubated with 3% hydrogen peroxide for 10 min, and diluted AURKB antibody (1:200 dilution) was added dropwise to the tissue of the slides for 24 h. The sections were washed, and then incubated for 20 min with the working solution of the secondary antibody dropwise at room temperature, and then incubated for 20 min with the secondary antibody dropwise. After rinsing with PBS buffer, DAB colour development and haematoxylin re‐staining were performed, and the slides were sealed after dehydration. Interpretation of staining: The intensity of staining and the proportion of stained cells were determined. Interpretation criteria: the intensity of staining was 0 points for no staining, 1 point for light yellow, 2 points for dark yellow and 3 points for yellow‐brown; the proportion of stained cells was scored: the percentage of stained cells in each section was randomly taken from five 400× fields of view. 0 was 0 points, 1% ~ 25% was 1 point, 26% ~ 50% was 2 points, 51% ~ 75% was 3 points and 76% ~ 100% was 4 points. The degree of positivity was determined based on the sum of the two results: <2 was negative; ≥2 was positive.

### 2.11 Statistical analysis

The variations in AURKB expression levels between cancerous and healthy tissues were assessed using the t‐test or the Wilcoxon rank‐sum test. Event‐survival curves were estimated by the Kaplan–Meier method, and survival analysis was conducted using the univariate Cox regression model. The Spearman test was employed for correlation analysis. Differences were considered statistically significant at *p* < 0.05.

## 3 RESULTS

### 3.1 Expression of AURKB mRNA and protein in various cancers

**FIGURE 2:**
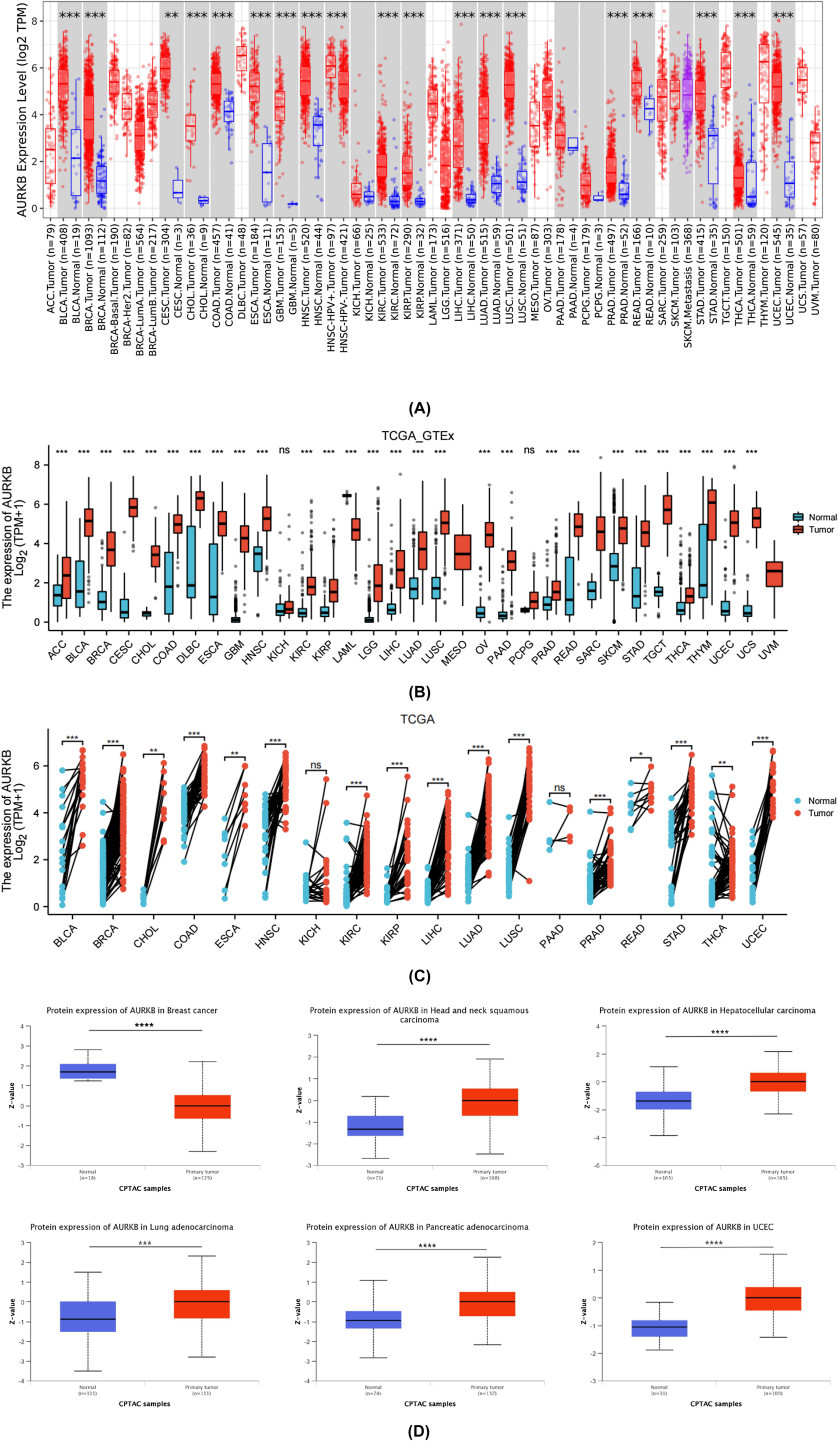
Expression of AURKB gene and protein in pan‐cancer (A) expression of AURKB mRNA in cancer in TIMER 2.0 database; (B) expression of AURKB mRNA in pan‐cancer in TCGA_GTEx samples;(C) expression of AURKB in pan‐cancer in paired samples from TCGA data; (D) Expression of AURKB protein in pan‐cancer. AURKB, Aurora kinase B; TIMER, Tumour Immunity Estimation Resource.

The results from the TIMER 2.0 database demonstrated that AURKB mRNA was overexpressed in 18 types of tumours, including BLCA, BRCA, COAD, CESC, CHOL, HNSC, ESCA, GBM, KIRC, LUSC, KIRP, LIHC, PRAD, LUAD, THCA, READ, STAD and UCEC (Figure 2A). An analysis of the TCGA_GTEx data samples revealed an elevated expression of AURKB mRNA in ACC, DLBC, LGG, OV, PAAD, SKCM, UCS, THYM and TGCT, compared to normal tissues, but excluding the aforementioned 18 types of cancer. Conversely, AURKB mRNA expression was found to be decreased in LAML tissues (Figure 2B). Furthermore, the study examined variations in AURKB expression among 18 cancer‐paired samples obtained from the TCGA dataset (Figure 2C). Findings from the CPTAC database indicated that cancer tissues of BRCA, HNSC, LIHC, LUAD, PAAD and UCEC exhibited notably elevated AURKB protein expression in comparison to normal tissues (Figure 2D).

### 3.2 Diagnostic significance of AURKB in various cancers

**FIGURE 3:**
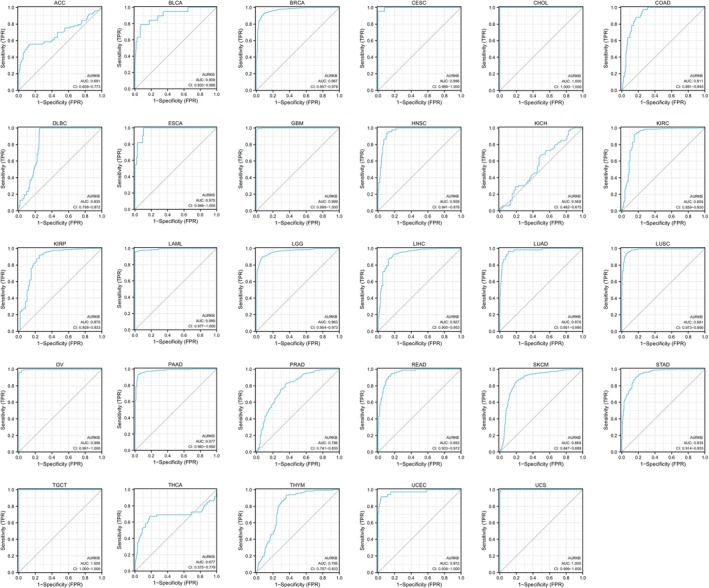
Diagnostic ROC curves for AURKB in pan‐cancer (AUC >0.9 considered highly accurate; 0.9 > AUC >0.7 considered with some accuracy; 0.7 > AUC >0.5 considered with lower accuracy). AUC, area under the cure; AURKB, Aurora kinase B; ROC, receiver operating characteristic.

After a thorough analysis of the diagnostic value of AURKB in malignancies, it was determined that AURKB exhibits remarkable precision in diagnosing 21 different cancers, namely BLCA, BRCA, CESE, CHOL, HNSC, COAD, ECSA, GBM, LIHC, KIRC, LAML, LGG, LUAD, LUSE, OV, PAAD, READ, TGCT, STAD, UCEC and USC, with the AUC value exceeding 0.9. The accuracy was found to be the highest for CHOL, TCGT and UCS, attaining an area under the curve of 1 (Figure 3).

### 3.3 Pan‐cancer examination of changes in AURKB methylation levels and genetic modifications

**FIGURE 4:**
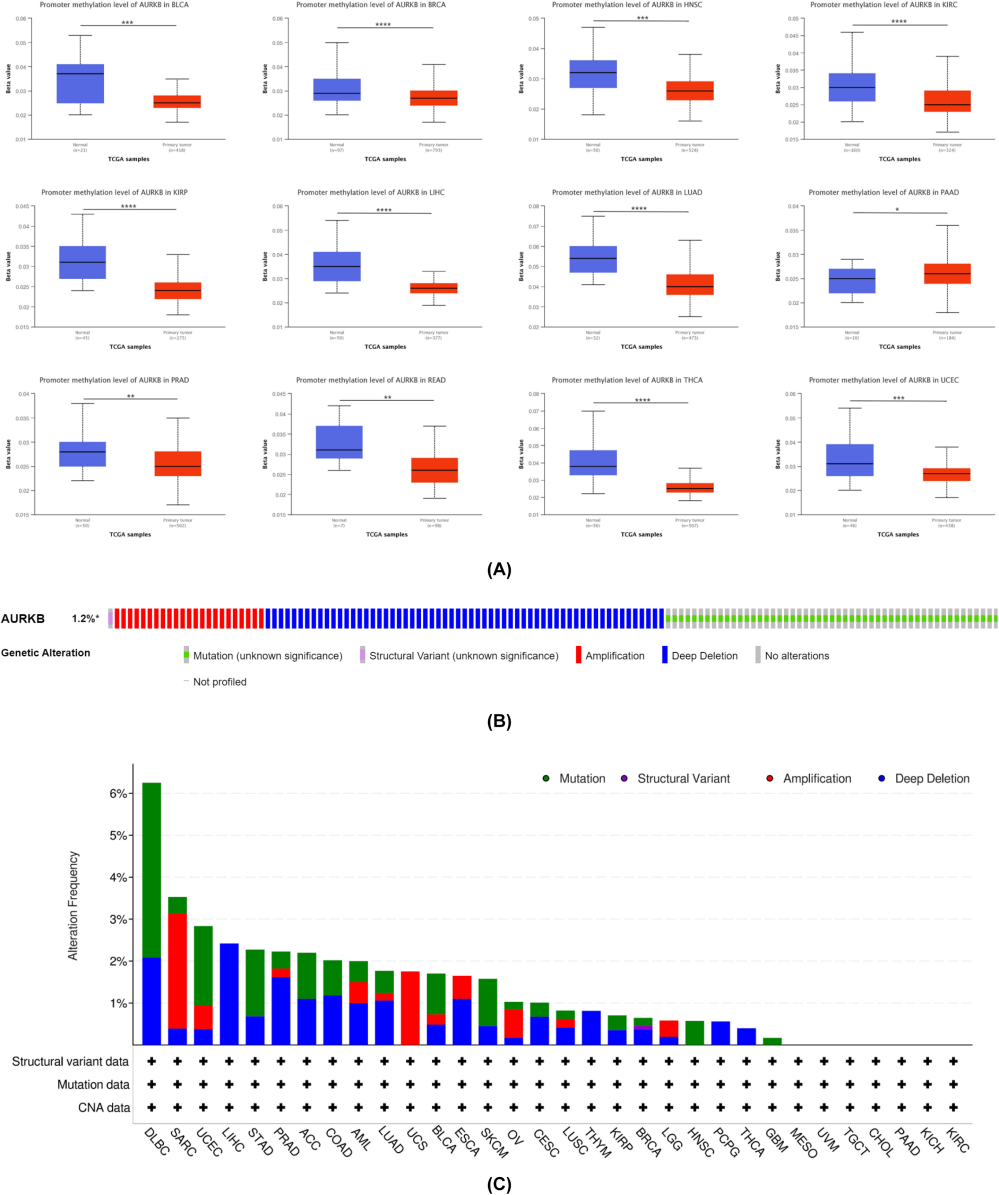
Promoter methylation and genetic alterations of AURKB in pan‐cancer (A) promoter methylation levels of AURKB in pan‐cancer; (B) and (C) mutation types and frequencies of AURKB in pan‐cancer in the cBioPortal database. AURKB, Aurora kinase B.

Gene promoter methylation has a direct impact on gene expression levels. In this study, the TCGA module within the UALCAN database was utilized to investigate the methylation of the AURKB promoter. The analysis revealed a decrease in AURKB methylation levels in BLCA, BRCA, HNSC, KIRC, KIRP, READ, LIHC, LUAD, PRAD, THCA and UCEC tissues in comparison to normal tissues. Conversely, PAAD tissues showed hypermethylation of the AURKB gene promoter (Figure 4A). Through the analysis of the cBioPortal online database, it was found that AURKB predominantly exhibited missense mutations, deep deletions and amplifications. The results indicate that AURKB mutations are commonly observed in DLBC, SARC, UCEC, LIHC, STAD, PRAD, ACC, COAD, AML, LUAD, UCS, BLCA, ESCA, SKCM, OV, CESC, LUSC, THYM, KIRP, BRCA, LGG, HNSC, PCPG, LUAD, UCS, BLCA, ESCA, SKCM, THCA and GBM (Figure 4B,C).

### 3.4 Prognostic significance of AURKB in various cancers

**FIGURE 5:**
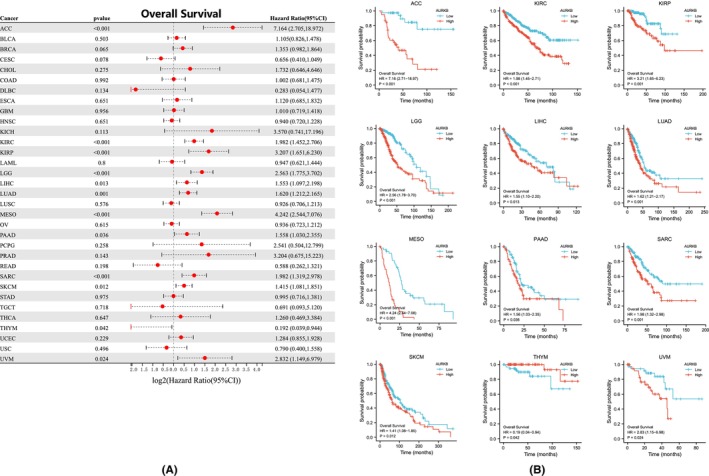
Relationship between AURKB expression and OS in pan‐cellular carcinomas (A) Forest plot of the risk ratio of AURKB expression and OS in pan‐cellular carcinomas; (B) Kaplan–Meier survival curves of AURKB expression on OS in ACC, KIRC, KIRP, LGG, LIHC, LUAD, MESO, PAAD, SARC, SKCM and UVM, respectively. ACC, adrenocortical carcinoma; AURKB, Aurora kinase B; KIRC, kidney renal clear cell carcinoma; KIRP; kidney renal papillary cell carcinoma; LGG, lower grade glioma; LIHC, liver hepatocellular carcinoma; LUAD, lung adenocarcinoma; MESO, Mesothelioma; OS, overall survival; PAAD, pancreatic adenocarcinoma; SARC, sarcoma; SKCM, skin cutaneous melanoma; UVM, uveal melanoma.

**FIGURE 6:**
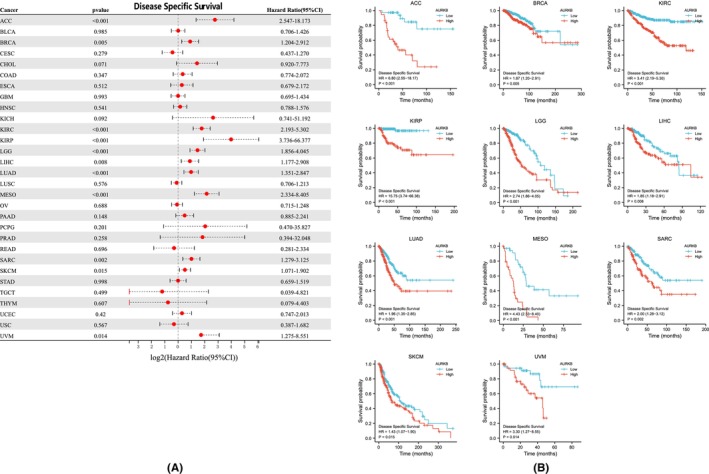
Association of AURKB expression with DSS in pan‐cellular carcinomas (A) Forest plot of the risk ratio of AURKB expression to DSS in pan‐cellular carcinomas; (B) Kaplan–Meier survival curves of AURKB expression on DSS in ACC, BRCA, KIRC, KIRP, LGG, LIHC, LUAD, MESO, SARC, SKCM and UVM. ACC, adrenocortical carcinoma; AURKB, aurora kinase B; BRCA, breast invasive carcinoma; DSS, disease‐specific survival; KIRC, kidney renal clear cell carcinoma; KIRP; kidney renal papillary cell carcinoma; LGG, lower grade glioma; LIHC, liver hepatocellular carcinoma; LUAD, lung adenocarcinoma; MESO, Mesothelioma; SARC, sarcoma; SKCM, skin cutaneous melanoma; UVM, uveal melanoma.

The relationship between AURKB expression and prognosis across multiple malignancies was explored through Cox regression analysis. AURKB expression was specifically found to be associated with OS in twelve distinct cancers: ACC, LIHC, KIRC, LUAD, KIRP, LGG, SKCM, MESO, THYM, PAAD, SARC and UVM (Figure 5A). Kaplan–Meier survival curves further indicated significant associations between elevated AURKB expression and unfavourable OS in ACC, LGG, KIRP, KIRC, LIHC, MESO, UVM, LUAD, SARC, PAAD and SKCM (Figure 5B). Investigation into the effect of AURKB expression on DSS revealed its influence on ACC, BRCA, LIHC, KIRC, KIRP, LGG, SARC, LUAD, MESO, UVM and SKCM (Figure 6A). Within these 11 malignancies, elevated AURKB expression was strongly correlated with a reduction in DSS (Figure 6B).

**FIGURE 7:**
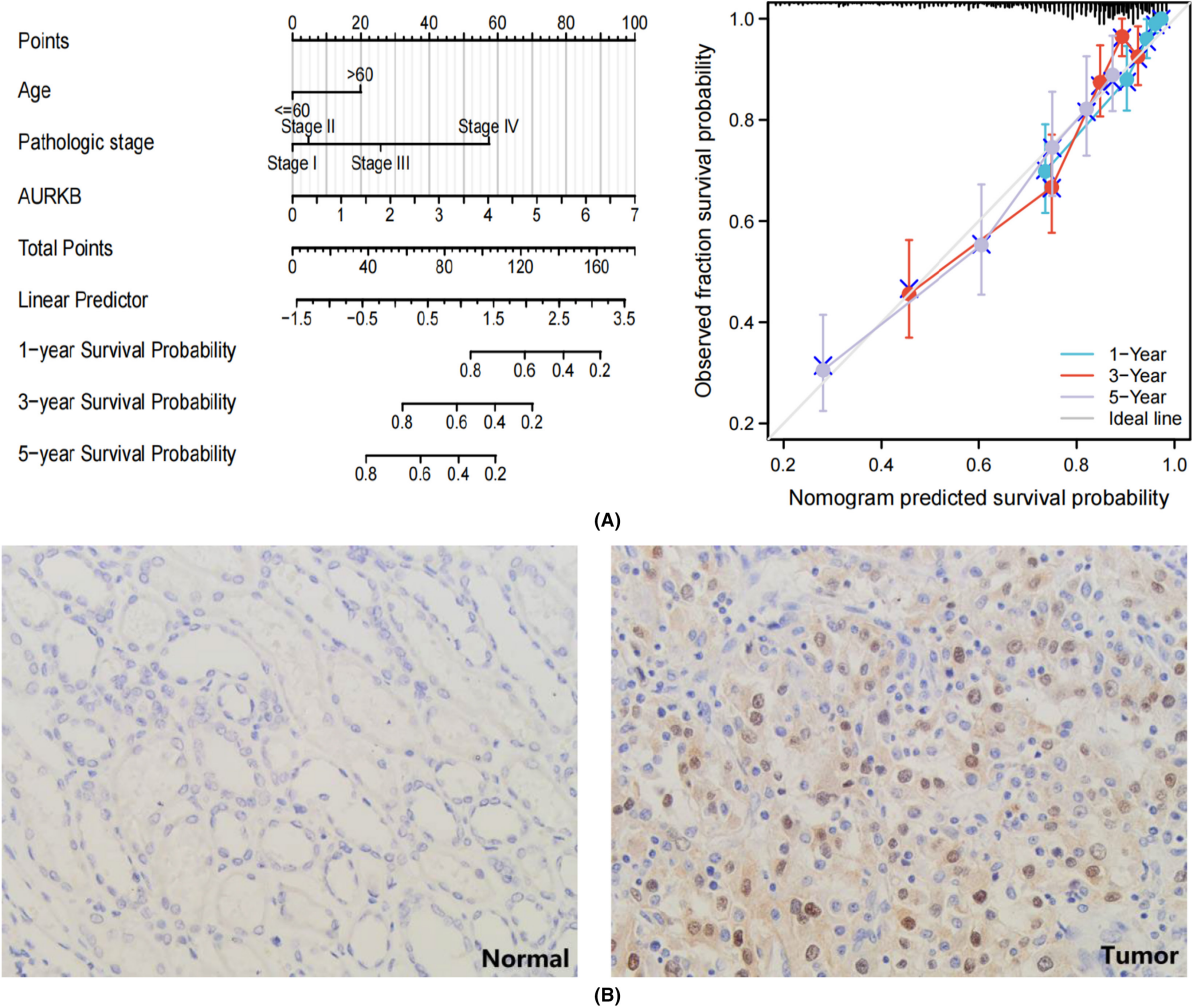
Establishment and validation of nomogram models based on KIRC data (A) Nomogram based on age, pathologic stage and expression of AURKB; Corrective analysis of column‐line diagrams. (B) Representative immunohistochemical analysis of AURKB in KIRC. AURKB, Aurora kinase B; KIRC, kidney renal clear cell carcinoma.

In an examination of the relationship between AURKB expression and the prognosis of specific cancer types, a risk prediction model for OS in KIRC was developed. This model incorporated datasets containing more than 500 KIRC samples. The analysis began by submitting clinical characteristics and AURKB expression from the KIRC dataset to both univariate and multivariate Cox regression analyses (Table 1). The model's prediction accuracy and calibration curves were evaluated by including independent prognostic factors derived from the analytical results. The findings demonstrate that AURKB serves as a robust predictive indicator for KIRC OS, with calibration curves reflecting the model's strong accuracy in predicting KIRC survival at 1‐year, 3‐year and 5‐year intervals (Figure 7A). To further verify the expression of AURKB in KIRC, we performed IHC experiments to observe the expression level of AURKB protein in KIRC tissues. The results showed that AURKB protein expression was mainly located in the nucleus, and the expression level of its protein was higher in cancer tissues (Tumour vs Normal = 21/50 versus 7/50, *p* = 0.002) (Figure 7B).

**TABLE 1:** Univariate and multivariate Cox regression analysis of KIRC patients.

Characteristics	Total(N)	Univariate analysis		Multivariate analysis	
		Hazard ratio (95% CI)	*p* Value	Hazard ratio (95% CI)	*p* Value
Age	539				
≤ 60	269	Reference			
> 60	270	1.765 (1.298–2.398)	< 0.001	1.655 (1.213–2.257)	0.001
Gender	539				
Female	186	Reference			
Male	353	0.930 (0.682–1.268)	0.648		
Pathologic stage	536				
Stage I	272	Reference			
Stage II	59	1.207 (0.650–2.241)	0.551	1.100 (0.590–2.052)	0.765
Stage III	123	2.705 (1.800–4.064)	< 0.001	1.888 (1.229–2.903)	0.004
Stage IV	82	6.692 (4.566–9.808)	< 0.001	4.340 (2.802–6.720)	< 0.001
Histologic grade	531				
G1	14	Reference			
G2	235	7510356.751 (0.000–Inf)	0.994	3319570.909 (0.000–Inf)	0.992
G3	207	14161426.542 (0.000–Inf)	0.993	4576085.139 (0.000–Inf)	0.992
G4	75	38204822.146 (0.000–Inf)	0.993	6756066.301 (0.000–Inf)	0.991
AURKB	539				
Low	269	Reference			
High	270	1.982 (1.452–2.706)	< 0.001	1.386 (1.000–1.922)	0.030

Abbreviations: AURKB, Aurora kinase B; KIRC, kidney renal clear cell carcinoma.

### 3.5 Correlation between AURKB expression and pathological stage in pan‐cancer

**FIGURE 8:**
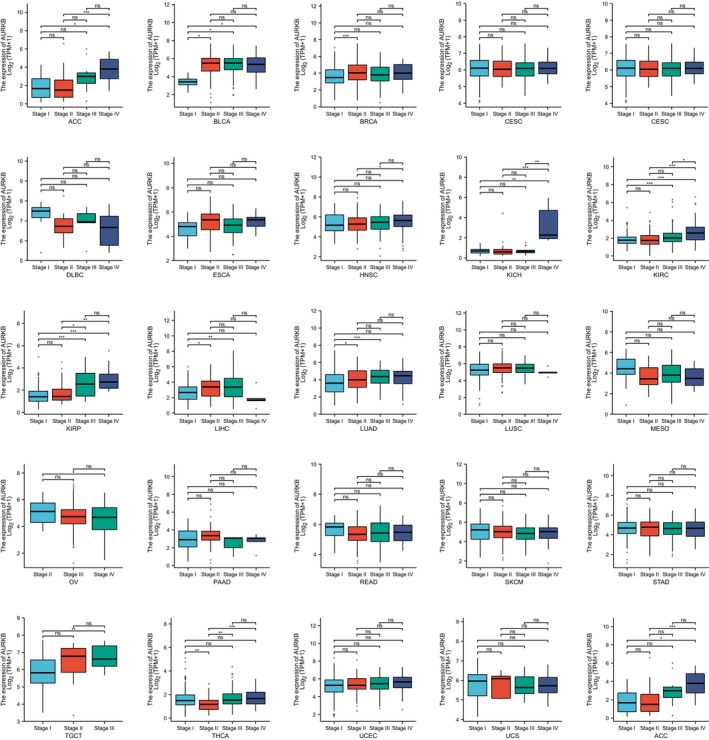
Correlation of AURKB with pathological staging of pan‐cancer. AURKB, Aurora kinase B.

Upon examination of AURKB expression across various pathological stages of pan‐cancer, it was found that AURKB was significantly upregulated in advanced stages of ACC, THCA, BLCA, KICH, BRCA, KIRC, LUAD, KIRP, LIHC and TGCT (Figure 8). These findings suggest that an increase in AURKB levels could be a potential marker for the progression of these particular cancer types.

### 3.6 Biological functions of AURKB in various cancers

**FIGURE 9:**
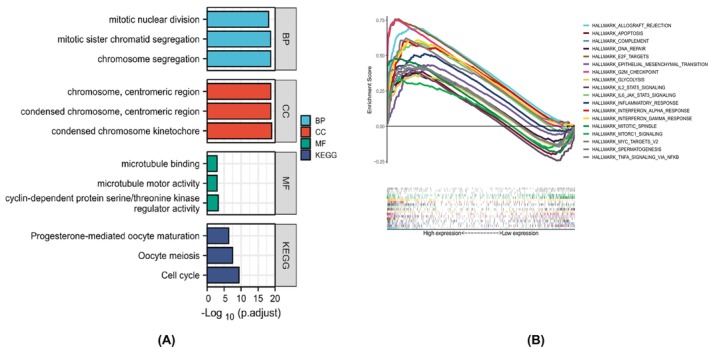
AURKB‐associated functional enrichment analyses (A) Enrichment analyses based on AURKB‐associated genes, including BP, CC, MF and KEGG; (B) The enriched gene sets of AURKB high‐expression group. AURKB, Aurora kinase B; BP, Biological processes; CC, cellular component; MF, molecular function.

To further explore the molecular role of AURKB in malignancies, an analysis was conducted using the KEGG and GO on the top 20 genes associated with AURKB, acquired from the STRING database. According to the GO analysis, genes associated with AURKB might be involved in the separation of sister chromatids during mitosis, nuclear division, the cell cycle, meiosis and various other biological processes (BP). The cellular component encompasses the chromosome, centromere, messome, microtubule and other components. Molecular function includes cyclin‐dependent serine/threonine kinase regulatory activity, and also encompasses microtubule movement and binding. The KEGG pathway enrichment analysis revealed that genes linked with AURKB are involved in a diverse array of BP such as cell cycle, human T‐cell leukaemia virus infection, cell senescence, oocyte meiosis, the P53 signalling pathway, progesterone‐mediated oocyte maturation, the FoxO signalling pathway, hepatitis B, viral carcinogenesis, human immunodeficiency virus type 1 infection and apoptosis across various species (Figure 9A).

The subsequent analysis performed using HALLMARK for GSEA demonstrated that increased AURKB expression was predominantly associated with numerous oncogenic pathways. These include allograft rejection, complement activation, programmed cell death, cell division, G2/M checkpoint regulation, DNA repair, the epithelial‐mesenchymal transition (EMT), glycolysis, immune response, targets of mcy, IL‐6/JAK/STAT3 signalling, interferon‐α response, IL‐2/STAT5 signalling, interferon‐γ response, sperm production, inflammatory response, mTORC1 signalling and TNF‐α/NF‐κB signalling (Figure 9B).

### 3.7 AURKB expression and immune cell infiltration

**FIGURE 10:**
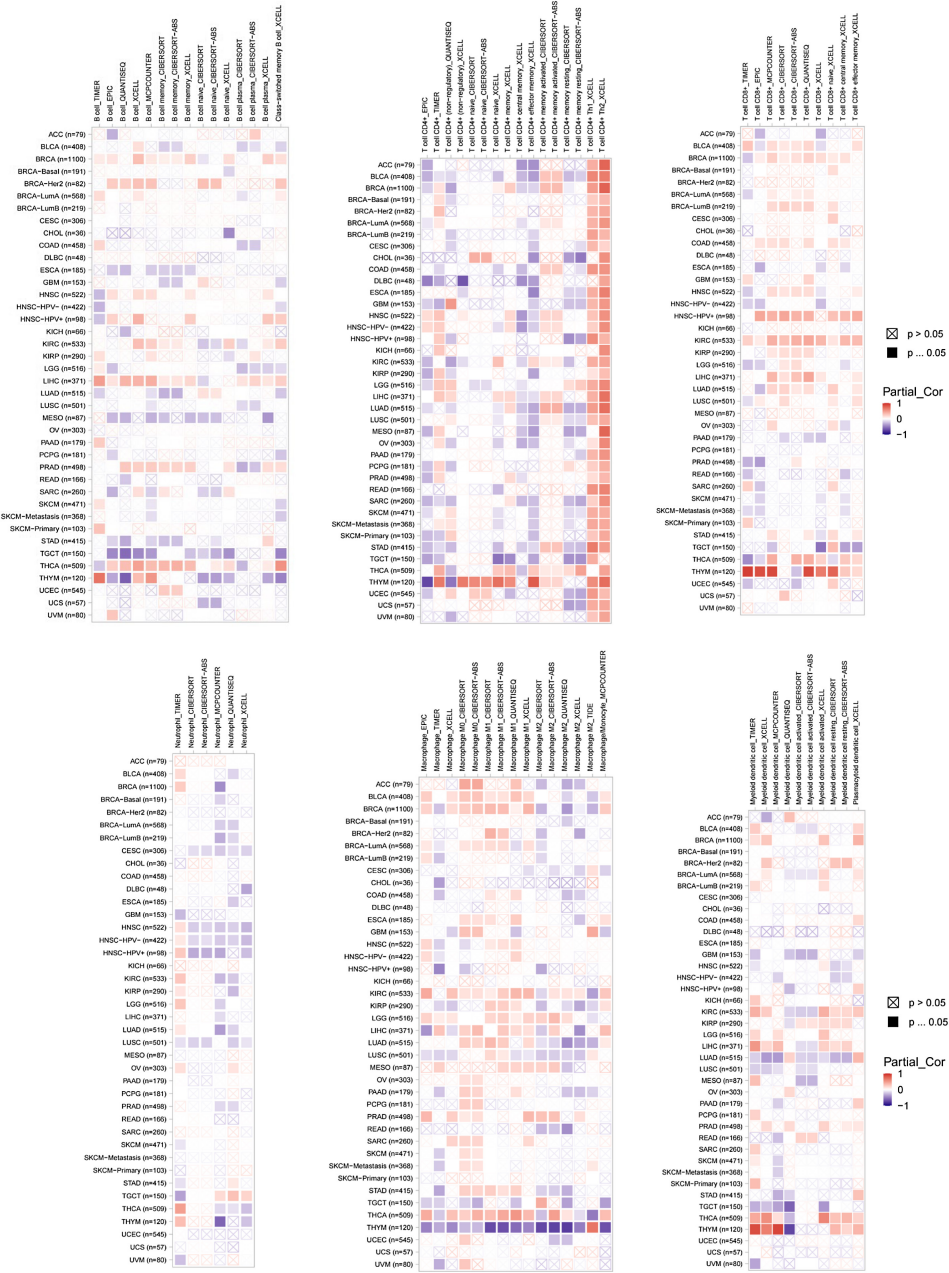
Relationship between AURKB expression and immune cell infiltration (heatmap of correlation between AURKB expression and B cells, CD4^+^ T cells, CD8^+^ T cells, neutrophils, macrophages and dendritic cells, respectively). AURKB, Aurora kinase B.

Immune cells play pivotal roles in tumorigenesis and development. In this study, the TIMER 2.0 database was employed to analyse the correlation between AURKB expression and the levels of immune cell infiltration in various cancers (Figure 10). The results were depicted as heat maps, revealing that different algorithms could yield varying outcomes. Notably, AURKB expression was positively correlated with CD4^+^ Th (T‐helper) 2 cells in all cancers except TGCT.

### 3.8 AURKB expression and immune‐related genes

**FIGURE 11:**
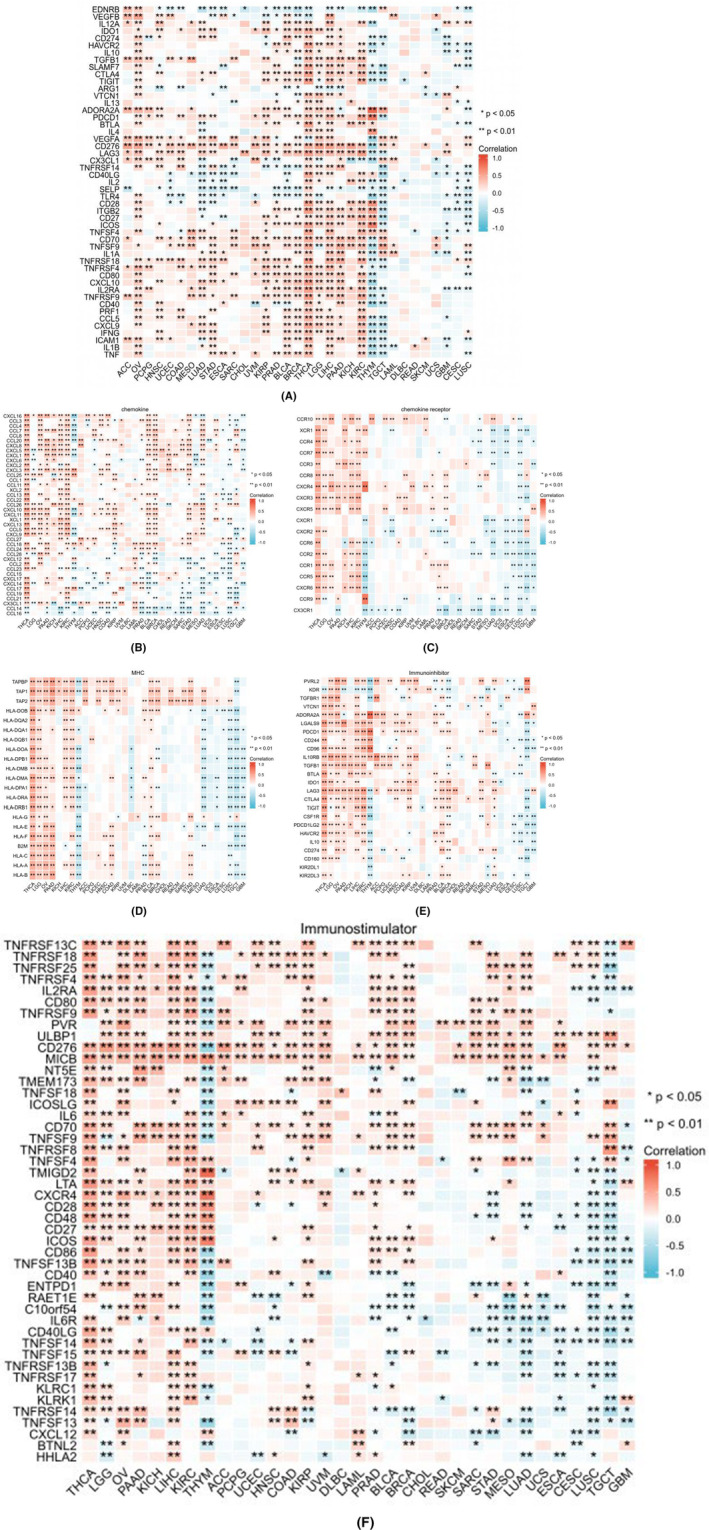
Correlation of AURKB expression with immune checkpoints and immunomodulation‐related genes in pan‐cancer (correlation between AURKB expression and immune checkpoints (A), chemokines (B), chemokine receptors (C), MHC (D), immunosuppressants (E) and immunostimulants (F)). AURKB, Aurora kinase B.

In specific cancer types, including OV, HNSC, LUAD, STAD, KIRP, PRAD, BLCA, BRCA, THCA, LGG, LIHC, PAAD and KIRC, the expression of AURKB was found to be positively associated with the majority of immune checkpoint genes (Figure 11A). Conversely, THYM, TGCT, CECS and LUSC exhibited a notable inverse relationship. Additionally, AURKB expression was positively correlated with the majority of immune factors, immune factor receptors, MHC, immunosuppressive genes and immunostimulatory genes in THCA, LGG, OV, PAAD, KICH, LIHC and KIRC. While AURKB demonstrated an inverse association with immunomodulatory genes in YHM, LUAD, ESCA, LUSC, TGCT and GBM, other cancers exhibited minimal or statistically insignificant connections with regulatory genes (Figure 11B–F).

### 3.9 Relationship between AURKB expression and TMB, or MSI

**FIGURE 12:**
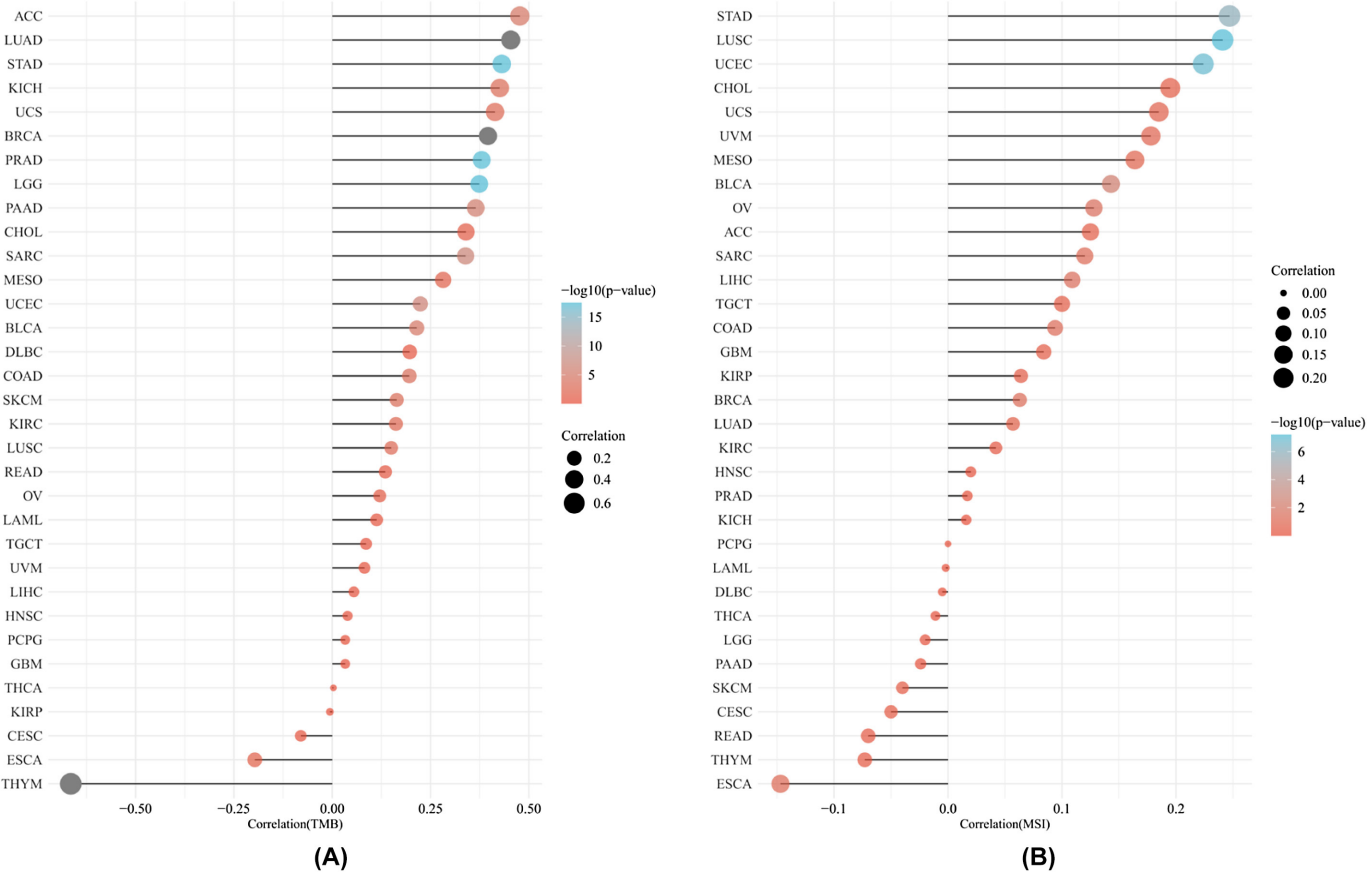
Correlation of AURKB expression with TMB and MSI in pan‐cancer (A) Relationship between AURKB expression and TMB in pan‐cancer; (B) Relationship between AURKB expression and MSI in pan‐cancer. AURKB, Aurora kinase B; MSI, microsatellite instability; TMB, tumour mutational burden.

The response to immunotherapy has been found to correlate with two emerging biomarkers: TMB and MSI. Our findings indicate that AURKB expression is positively correlated with the TMB in a variety of cancers, including ACC, BLCA, BRCA, CHOL, COAD, KICH, KIRC, LGG, LUAD, LUSC, MESO, PAAD, SARC, PRAD, SKCM, STAD, UCS and UCEC. However, negative correlation was observed in THYM and ESCA (Figure 12A). Additionally, elevated AURKB levels were linked with increased MSI in BLCA, BRCA, LIHC, LUSC, OV, STAD and UCEC, with no correlation observed in other types of cancer (Figure 12B).

### 3.10 Target miRNA Prediction

**FIGURE 13:**
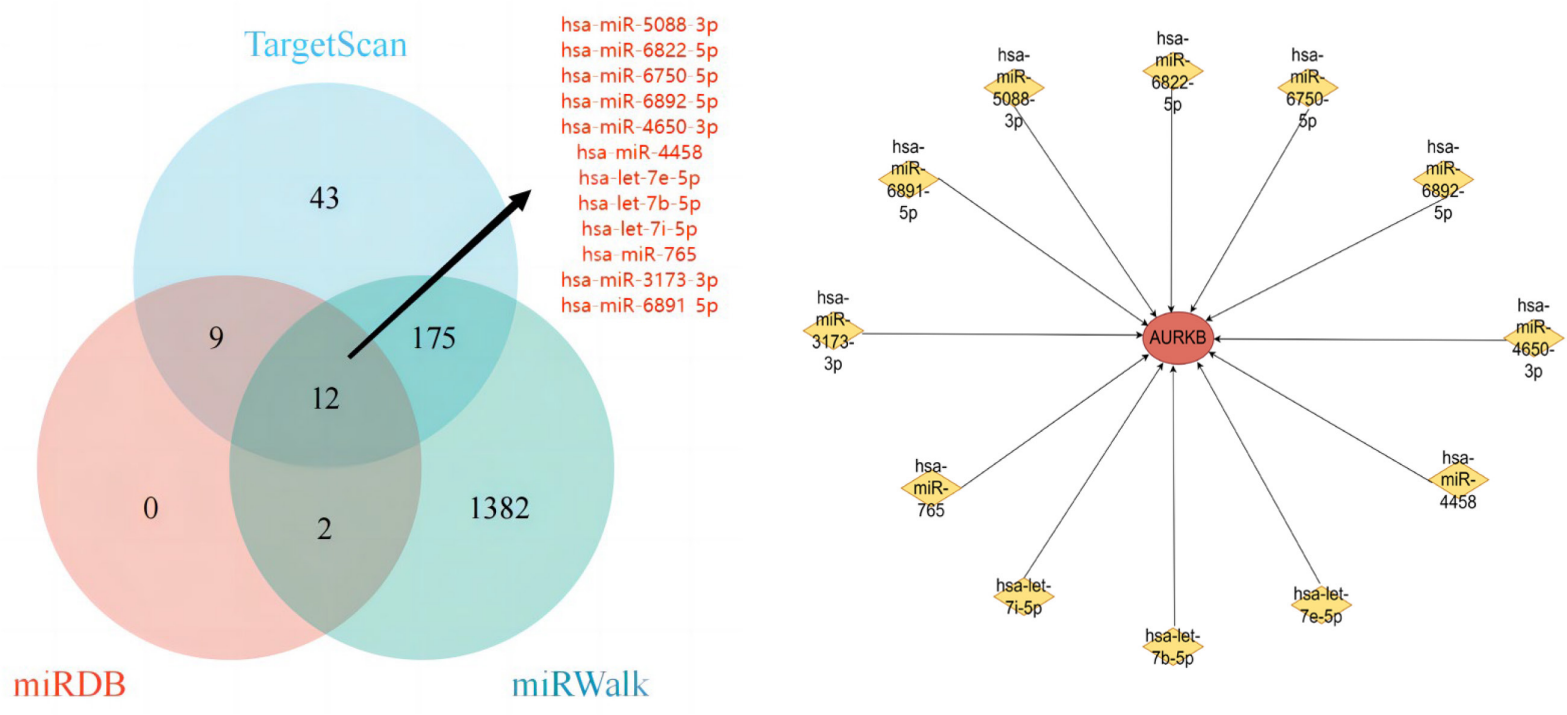
miRNA‐mRNA network of AURKB. AURKB, Aurora kinase B.

miRNAs can induce gene silencing and down‐regulate gene expression by binding to mRNAs. We firstly obtained 23, 250, and 1571 target miRNAs of AURKB from miRDB, TargetScan and miRWalk databases respectively, and finally obtained 12 important target miRNAs, which are hsa‐miR‐5088‐3p, hsa‐miR‐6822‐5p, hsa‐miR‐6750‐5p, hsa‐miR‐6892‐5p, hsa‐miR‐4650‐3p, hsa‐miR‐4458, hsa‐let‐7e‐5p, hsa‐let‐7b‐5p, hsa‐let‐7i‐5p, hsa‐miR‐765, hsa‐miR‐3173‐3p, hsa‐miR‐6891‐5p. we then built a network diagram of AURKB‐associated miRNA‐mRNAs based on the obtained data (Figure 13).

## 4 DISCUSSION

In this study, we determined that AURKB is widely overexpressed in cancer and is significantly associated with poorer prognosis in cancer patients, and can be used as a diagnostic and prognostic marker for a variety of cancers. Most importantly, we found that AURKB is significantly associated with immune cell infiltration, immune factors, immune checkpoints, TMB and MSI, which can effectively predict the response to cancer immunotherapy. Our findings provide important clues for further investigation of the potential role of AURKB in tumour immunity and immunotherapy.

This research marks the first investigation into the correlation between AURKB and pan‐cancer. A detailed examination of AURKB mRNA expression across various malignancies was conducted utilizing TCGA data, TCGA_GTX data and TCGA matched samples. It was revealed that AURKB exhibits increased expression in the majority of malignancies in comparison to regular tissues. AURKB's expression indicated a positive diagnostic significance for multiple cancer types. The AUC exceeded 0.7 in 26 types of cancers and surpassed 0.9 in 21 types. This study's findings from the analysis of Cox regression and Kaplan–Meier demonstrate a robust correlation between increased AURKB expression and reduced OS and DSS among individuals diagnosed with ACC, KIRP, BRCA, CHOL, KIRC, MESO, LGG, LIHC, LUAD, PAAD, THYM, SARC, SKCM and UVM. Previous studies have highlighted that the expression of AURKB is connected to the prognosis of malignancies such as NSCLC^12^ and prostate cancer.^13^ For instance, Lin's research revealed a significant upregulation of AURKB in NSCLC tissues, which was correlated with decreased OS and PFS. Similarly, Hongo observed elevated AURKB expression in prostate cancer tissues compared to adjacent normal tissues, with higher expression levels associated with a negative impact on recurrence‐free survival prognosis. Furthermore, the study revealed that heightened AURKB expression is associated with decreased sensitivity of DU145 cells to carbataxel, suggesting that AURKB overexpression plays a role in the acquisition of resistance to this therapeutic agent. Recent investigations have also demonstrated that AURKB is overexpressed in retinoblastoma compared to neighbouring healthy retinas, and its expression correlates significantly with histological risk factors including invasion of the optic nerve and anterior chamber.^14^ Beyond the 33 typical cancers, AURKB displays notable expression in various rare tumours, suggesting a potential association with an unfavourable prognosis. This highlights the need for further studies to corroborate this hypothesis.

Genetic and epigenetic modifications exert a substantial impact on gene expression. A notable mechanism of epigenetic heredity entails DNA modification via methylation.^15^ Various investigations have demonstrated that alterations in the methylation status of a gene's promoter region can result in either suppression or upregulation of the gene's expression.^16^ Through an examination of the relationship between AURKB promoter methylation and cancer, it was observed that AURKB expression increased as the methylation level decreased in various types of cancer, including BLCA, BRCA, HNSC, LUAD, KIRC, KIRP, LIHC, THCA, PRAD, READ and UCEC. This finding suggests that the elevated expression of AURKB may be linked to epigenetic modification.

The analysis of 20 genes closely related to AURKB revealed that the functional enrichment within the framework of Gene Ontology is intimately connected to the cell cycle, emerging as the leading pathway in the KEGG database. This observation underscores that the potential cancer‐inducing attributes of AURKB might be realized through its influence on the process of cellular division. Previous investigations have demonstrated that AURKB interacts with CCND1 and cyclin K, both proteins significantly related to the cell cycle, in the context of gastric cancer^17^ and prostate cancer,^18^ respectively. Additionally, it has been observed that genes related to AURKB are enriched in cell aging and P53 signalling pathways. While cell senescence has traditionally been understood as a mechanism for suppressing tumours, recent research has shown that aged cancer cells can actually promote proliferative signals, evade programmed cell death, induce angiogenesis, expedite invasion and metastasis and hinder tumour immunity through various mechanisms.^19^, ^20^ It is a widely accepted notion that a reduction in P53 gene activity can be linked to tumorigenesis and advancement.^21^ In alignment with the GSEA analysis, it has been discovered that elevated levels of AURKB correspond with pathways related to the cell cycle (including apoptosis, mitosis, G2/M checkpoint,^22^, ^23^ DNA repair,^22^ MGY‐V2 target^24^, ^25^), pathways intrinsic to inflammation (such as inflammatory response,^26^ IL‐6/JAK/STAT3 signalling pathway,^27^, ^28^ IL‐2/STAT5 signalling pathway,^29^ TNF‐α/NF‐κB signalling pathway^30^), pathways pertinent to the immune system (like allograft rejection, complement, mTORC1 signalling pathway,^31^, ^32^ IFN‐α response,^33^ IFN‐γ response^34^) and EMT.^35^, ^36^ These discoveries present a plausible association between AURKB overexpression and both tumorigenesis and development.

In the complex milieu of the tumour microenvironment, immune cells play a pivotal role in modulating tumour growth and prognosis.^37^, ^38^, ^39^ The findings of this study indicate a positive correlation between the expression of AURKB and the level of CD4^+^ Th2 cells in various types of cancers, with the exception of TGCT. Th1 and Th2 cells, as major subpopulations of CD4^+^ T cells, exhibit distinct differentiation pathways, with Th1 cells primarily influenced by IL‐12 and IFN‐γ, and Th2 cells associated with IL‐4. Th1 cells are known for their role in the production of IFN‐γ, IL‐2 and TNF‐α, among other cytokines, making them the primary Th cell subset involved in the anti‐tumour immune response.^40^ Conversely, Th2 cells have been demonstrated to inhibit the immune system and facilitate tumour growth and spread. Numerous studies have indicated an imbalance in Th cell subsets in cancer patients, with Th2 cells predominantly found at tumour sites and Th1 cells predominantly found in non‐tumour tissues.^41^ Therefore, AURKB may play a role in tumorigenesis by promoting the secretion of IL‐4 from CD4^+^ T cells, resulting in the excessive differentiation of Th2 cells and dysregulation of Th cell subpopulations. Notably, AURKB expression displayed strong correlations with TMB and MSI. These findings imply the potential for enhancing the effectiveness of cancer immunotherapy through targeting AURKB.

MiRNAs can induce gene silencing and down‐regulate gene expression by binding to mRNAs. Therefore, identifying cancer‐associated miRNAs and establishing related miRNA‐mRNA networks are essential for further cancer research. While this study only used an online database for preliminary screening of target miRNAs for AURKB, it did not involve complex and accurate algorithms and models to improve the accuracy and performance of miRNA identification, which is lacking in this study. Wang introduced a novel framework, termed ‘Rotation Forest for Essential MicroRNA identification (RFEM),’ for the prediction of miRNA characteristics in mice. The incorporation of miRNA functional attributes into the model served to enhance the variety of miRNA features. The utilization of a rotation forest model with decision trees as base classifiers, coupled with feature transformation to amplify distinctions between the base classifiers, yielded improved ensemble outcomes. This work lays a foundation for the advancement of miRNA models pertinent to human cancer research.^42^ Huang showed that in the era of big data, machine learning can be scaled up to larger and richer datasets, including data from different populations, different cancer types and different disease stages. Effective feature selection methods are utilized in order to extract the most relevant features from large‐scale miRNA data. This may involve data dimensionality reduction techniques such as principal component analysis or feature selection algorithms such as filtering, packing or embedding based approaches. Different types of machine learning models such as Support Vector Machines, random forest, deep learning models are then passed through in order to find the most suitable model for solving the problem. In addition, the models are optimized to improve the performance such as hyperparameter tuning, integrated learning and so on. Among the various learning models, deep learning is due to other machine learning methods in terms of performance, but its interpretability is poor.^43^, ^44^, ^45^ Future directions may focus on how to make models more explanatory so that researchers can understand the predictions of the models and derive meaningful biological insights from them. Most importantly, in our view, the models developed need to be able to be applied in clinical practice. Therefore, future directions may include the development of miRNA diagnostic tools for specific cancer types or individualized treatments. This may involve clinical trials and validation in practice to ensure the validity and reliability of the models, further driving further development and application in the field.

### 4.1 Strengths and limitations

The strengths of our study are the exploration of the high level of expression of AURKB genes and proteins in cancer and the correlation with poor prognosis, as well as the role of AURKB in the immune pathway, in particular its potential application in cancer immunotherapy. This information may have potential applications for primary care physicians (PCPs). First, PCPs can improve their ability to make early diagnosis and prognostic assessment of patients by understanding the high level of expression of AURKB genes and proteins in a variety of cancers and their association with poor prognosis. They can be more alert to patients with cancer risk factors and adopt more aggressive strategies for screening and treatment at an early stage. Second, an understanding of the role of AURKB in the immune pathway could provide PCPs with new ideas for treatment. They can consider the use of targeted therapies against AURKB in conjunction with existing cancer treatment options to improve patient outcomes and survival. In addition, knowledge of the role of AURKB in the immune pathway could lead PCPs to better understand the rationale and potential scope of the application of immunotherapy, thereby better guiding treatment choices for patients. Finally, the developed survival prediction model for KIRC patients could provide PCPs with a tool to quickly and accurately assess the survival prediction of KIRC patients with positive AURKB expression. By utilizing this model, PCPs can better guide the direction of treatment for their patients and better assess treatment efficacy and prognosis.

Nevertheless, there exist certain limitations within this study. Initially, the research draws upon data gathered from publicly available repositories and primarily focuses on bioinformatics analysis, lacking supplementary validation of the findings at both the cellular and animal levels. Additionally, the underlying pathophysiological mechanisms have not been fully explored. Therefore, additional exploration is needed to fully understand the carcinogenic mechanism of AURKB and to evaluate its potential application as a therapeutic target.

## 5 CONCLUSION

Elevated expression of AURKB is strongly associated with an unfavourable prognosis in various types of tumours. A decrease in the methylation of AURKB has been observed across multiple cancer types, illustrating a critical link between this gene and malignant transformations. Furthermore, AURKB expression has been found to correlate significantly with the infiltration of immune cells, the presence of immunomodulatory genes, TMB and MSI within tumours. These findings collectively suggest that AURKB may hold substantial promise as a potential target for cancer immunotherapy, warranting further exploration and validation in future research.

## AUTHOR CONTRIBUTIONS

**Jun Li:** Methodology (equal); software (equal); writing – review and editing (equal). **Cui Cheng:** Data curation (equal); formal analysis (equal); resources (equal). **Jiajun Zhang:** Funding acquisition (equal); writing – review and editing (equal).

## FUNDING INFORMATION

This study was partially supported by the Key Programs of Natural Science Research in Colleges and Universities in Anhui Province, China (Grant Number: KJ2020A1290).

## CONFLICT OF INTEREST STATEMENT

The authors declare that the research was conducted in the absence of any commercial or financial relationships that could be construed as a potential conflict of interest.

## Data Availability

The datasets presented in this study can be found in online repositories. The names of the repository/repositories and accession number(s) can be found in the article material.
